# Biomarker Discovery for the Carcinogenic Heterogeneity Between Colon and Rectal Cancers Based on lncRNA-Associated ceRNA Network Analysis

**DOI:** 10.3389/fonc.2020.535985

**Published:** 2020-10-30

**Authors:** Xin Qi, Yuxin Lin, Xingyun Liu, Jiajia Chen, Bairong Shen

**Affiliations:** ^1^ School of Chemistry and Life Sciences, Suzhou University of Science and Technology, Suzhou, China; ^2^ Center for Systems Biology, Soochow University, Suzhou, China; ^3^ Department of Urology, The First Affiliated Hospital of Soochow University, Suzhou, China; ^4^ Institutes for Systems Genetics, West China Hospital, Sichuan University, Chengdu, China

**Keywords:** cancer heterogeneity, lncRNA biomarker, miRNA biomarker, ceRNA network, colon and rectal cancers, initiation and metastasis****

## Abstract

**Background:**

Colorectal cancer (CRC) is one of the leading causes of cancer death worldwide. Emerging evidence has revealed that risk factors and metastatic patterns differ greatly between colon and rectal cancers. However, the molecular mechanism underlying their pathogenic differences remains unclear. Therefore, we here aimed to identify non-coding RNA biomarkers based on lncRNA-associated ceRNA network (LceNET) to elucidate the carcinogenic heterogeneity between colon and rectal cancers.

**Methods:**

A global LceNET in human was constructed by employing experimental evidence-based miRNA-mRNA and miRNA-lncRNA interactions. Then, four context-specific ceRNA networks related to cancer initiation and metastasis were extracted by mapping differentially expressed lncRNAs, miRNAs and mRNAs to the global LceNET. Notably, a novel network-based bioinformatics model was proposed and applied to identify lncRNA/miRNA biomarkers and critical ceRNA triplets for understanding the carcinogenic heterogeneity between colon and rectal cancers. Moreover, the identified biomarkers were further validated by their diagnostic/prognostic performance, expression pattern and correlation analysis.

**Results:**

Based on network modeling, lncRNA KCNQ1OT1 (AUC>0.85) and SNHG1 (AUC>0.94) were unveiled as common diagnostic biomarkers for the initiation and metastasis of colon and rectal cancers. qRT-PCR analysis uncovered that these lncRNAs had significantly higher expression level in CRC cell lines with high metastatic potential. In particular, KCNQ1OT1 and SNHG1 function in colon and rectal cancers *via* different ceRNA mechanisms. For example, KCNQ1OT1/miR-484/ANKRD36 axis was involved in the initiation of colon cancer, while KCNQ1OT1/miR-181a-5p/PCGF2 axis was implicated in the metastasis of rectal cancer; the SNHG1/miR-484/ORC6 axis played a role in colon cancer, while SNHG1/miR-423-5p/EZH2 and SNHG1/let-7b-5p/ATP6V1F axes participated in the initiation and metastasis of rectal cancer, respectively. In these ceRNA triplets, miR-484, miR-181a-5p, miR-423-5p and let-7b-5p were identified as miRNA biomarkers with excellent distinguishing ability between normal and tumor tissues, and ANKRD36, PCGF2, EZH2 and ATP6V1F were closely related to the prognosis of corresponding cancer.

**Conclusion:**

The landscape of lncRNA-associated ceRNA network not only facilitates the exploration of non-coding RNA biomarkers, but also provides deep insights into the oncogenetic heterogeneity between colon and rectal cancers, thereby contributing to the optimization of diagnostic and therapeutic strategies of CRC.

## Introduction

Colorectal cancer (CRC) is one of the most commonly diagnosed malignant tumors with high prevalence and mortality rates worldwide. Tumor progression and distant metastasis are the major lethal factors for CRC patients, leading to 5-year survival rate less than 10% (1). Moreover, anatomical distinction between colon cancer and rectal cancer was closely related to patient morbidity, risk factor, therapeutic strategies, and especially may have an impact on prognosis (2–4). Therefore, systematically illuminating the molecular mechanisms and discovering predictive biomarkers specific respectively to colon and rectal cancers are in urgent need for the diagnosis and treatment of CRC.

Recent advances in high-throughput sequencing technology have uncovered that there is a tremendous number of RNAs without protein-coding potential, defined as non-coding RNAs (ncRNAs) (5). For example, microRNAs (miRNAs) are a class of well-characterized ncRNAs that post-transcriptionally regulate target gene expression through complementary pairing (6–8); long non-coding RNAs (lncRNAs) are a class of recently discovered ncRNAs longer than 200 nucleotides with emerging roles in diverse cancer-related processes, e.g., proliferation, invasion, metastasis, and metabolism (9, 10). Owing to the huge potential in regulating gene expression, considerable effort has been made to decode how ncRNAs exert functions in cancer-related biological processes. Salmena etal. (11) proposed a ceRNA (competing endogenous RNA) hypothesis, which states that RNA transcripts sharing common miRNA response elements (MREs), e.g. lncRNAs, pseudogenes, circular RNAs and competing mRNAs, can compete for miRNA binding and thereby reciprocally modulate each other’s expression. Increasing evidence has supported that miRNA-mediated ceRNA crosstalk is widely involved in the pathogenesis of multiple cancers (12), representing a novel layer of post-transcriptional gene regulation. Notably, mounting evidence has demonstrated that lncRNAs could act as oncogenic or tumor-suppressive genes in the initiation and progression of CRC through a ceRNA mechanism. For example, lncRNA SNHG7 was found to be significantly over-expressed in CRC. It accelerates tumor proliferation and metastasis by serving as a miR-216b-mediated ceRNA of GALNT1 (13). Thus, lncRNAs represent promising diagnostic biomarkers and therapeutic targets for CRC, thereby becoming the research hotspot among the ceRNA family.

As the perturbation of pivotal RNA abundance in the ceRNA network could lead to cancer initiation and/or progression, a growing number of researchers have made great effort to identify the lncRNA biomarkers in specific cancer *via* constructing lncRNA-associated ceRNA network (LceNET) (14, 15). Notably, the lnCeDB database providing a collection of human lncRNAs that potentially function as ceRNA was built in 2014 (16). However, it has not been updated continuously, resulting in inconsistency of lncRNA ID version between lnCeDB and TCGA database, which is a widely utilized pool of high-throughput cancer datasets. Those facts revealed that, optimizing the method of constructing ceRNA network and systematically evaluating lncRNA biomarkers that act as ceRNA are urgently needed to explore the molecular distinction in the pathogenesis of cancers.

**Figure 1:**
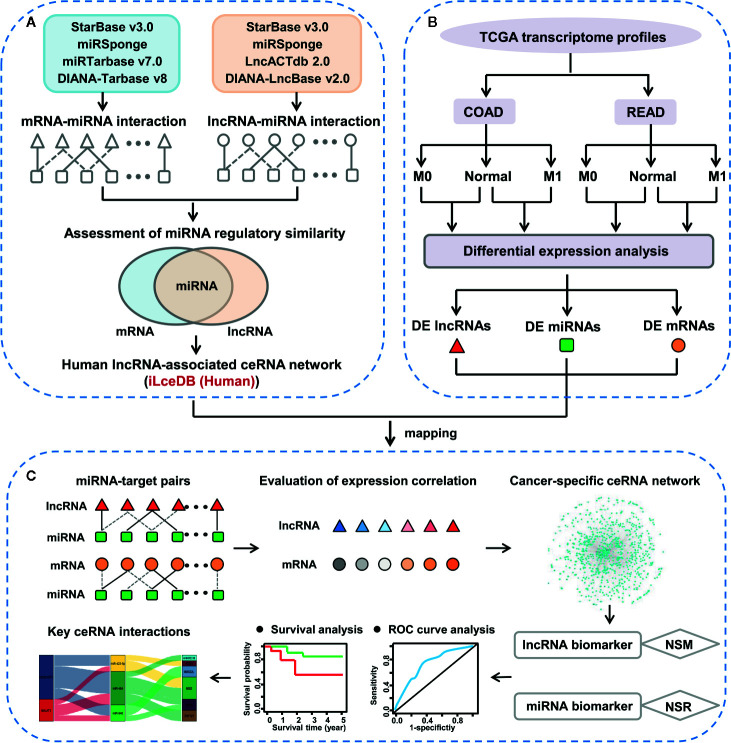
The integrative pipeline for ceRNA network construction and characterization. **(A)** Construction of lncRNA-associated ceRNA network in human. **(B)** Identification of lncRNAs, miRNAs and mRNAs with differential expression pattern between colon/rectal cancers and corresponding normal tissues. **(C)** Discovery of key ceRNA interactions based on context-specific LceNET network.

In this study, we focused on comparatively decoding molecular mechanism underlying the initiation and metastasis of colon and rectal cancers through a ceRNA network manner. To facilitate the investigation of context-specific ceRNA crosstalk, we firstly constructed a global LceNET in human and applied a computational approach to identify lncRNA/miRNA biomarkers through constructing LceNETs implicated in the initiation and metastasis of colon and rectal cancers. Moreover, key lncRNA-miRNA-mRNA interactions associated with pathogenesis were identified in colon and rectal cancers, respectively. The pipeline of the present study was shown in **Figure 1**. Thus, systematically identifying and comparing lncRNA biomarkers acting as ceRNAs, could contribute to elucidate the similarities and differences of the molecular mechanisms between colon and rectal cancers, thereby providing valuable clues for CRC therapy.

## Materials and Methods

### TCGA Data Collection

To explore ceRNA biomarkers associated with colorectal carcinogenesis, the level 3 RNA-Seq gene expression data, miRNA-seq data and clinical data of 459 colon cancer patients and 171 rectal cancer patients were retrieved from TCGA-COAD and TCGA-READ database (up to March 26, 2019), respectively. According to the tumor-node-metastasis (TNM) staging system of colorectal cancer, patients were divided into without distant metastasis (M0) or with distant metastasis (M1) subgroups. As adenocarcinoma is the most common histotype accounting for 90% of CRC cases (17), only adenocarcinoma cases with matched RNA-seq and miRNA-seq data were retained for subsequent analyses. The sample number of each group was shown in the **Supplementary Figure 1**. The study was performed according to the TCGA guidelines.

### Construction of the Global LceNET in Human

Based on “ceRNA hypothesis” (11), the lncRNA-associated competing triplets consist of miRNA-lncRNA and miRNA-mRNA interactions sharing at least one common miRNA. Therefore, identifying miRNA-target interactions is a prerequisite to recognize lncRNA-related ceRNA triplet. Accordingly, the global lncRNA-associated ceRNA network in human was constructed by the following two steps.

First, high-confidence miRNA-target interactions supported by low-/high-throughput experiments were collected and integrated. The experimentally verified miRNA-mRNA pairs were downloaded from starBase v3.0 (18), DIANA-Tarbase v8 (19), miRTarbase (v7.0) (20) and miRSponge (21) databases, while the experimentally validated miRNA-lncRNA pairs were extracted from starBase v3.0 (18), LncACTdb2.0 (22), DIANA-LncBase Experimental v.2 (23) and miRSponge (21) databases. Importantly, considering the unprecedented utility of TCGA database for data mining across cancers, lncRNAs were converted to be consistent with the annotation file of TCGA dataset (GENCODE v22). By integrating the interactions among above corresponding databases and removing redundant relationships, 718,708 miRNA-mRNA pairs and 29,373 miRNA-lncRNA pairs were finally obtained.

Second, to further identify competing lncRNA-mRNA interactions, a hypergeometric test was performed to evaluate the significance of shared common miRNAs between each lncRNA-mRNA pairs. The *P* was calculated as follows:

$$P=1-\sum_{i=0}^{x-1}\frac{\left(\underset{i_{}}{N_{lnc}}\right)\left(\underset{N_{mRNA}-i}{N_{T}-N_{lnc}}\right)}{\left(\underset{N_{mRNA}}{N_{T}}\right)}$$

where *N_T_* represents the total number of miRNAs in human genome, *N_lnc_* and *N_mRNA_* represent the total number of miRNAs that regulate lncRNA and mRNA, respectively, and *x* is the number of miRNAs shared by lncRNA and mRNA. The *P* was subject to Benjamini-Hochberg correction. The lncRNA-mRNA competing pairs with adj.*P*<0.01 were selected as significant pairs. Finally, 20,888,725 lncRNA-miRNA-mRNA pairs were selected as ceRNA interactions.

### Identification of Differentially Expressed lncRNAs, miRNAs, and mRNAs

To keep consistent with gene annotations in TCGA, lncRNAs and mRNAs in RNA-Seq expression data were firstly identified and annotated by using GENCODE database (v22) (24), respectively. Meanwhile, the accession number of miRNAs in miRNA-Seq data were converted into miRNA official symbol referring to miRBase database (v21) (25). RNAs which cannot be converted into gene symbol were excluded. Then, the count data were analyzed using edgeR package (26) to identify differentially expressed lncRNAs, mRNAs and miRNAs between normal and M0/M1 tissues. lncRNAs/mRNAs/miRNAs that sufficiently expressed (count per million (CPM) > 1) in at least 80% samples were kept in the differential expression analysis. Then, the lncRNAs/mRNAs/miRNAs were considered significantly differentially expressed based on the following criteria: |log_2_(fold change)| >=1 and false discovery rate (FDR) <0.05.

### Reconstruction of Context-Specific ceRNA Networks for Cancer Initiation and Metastasis

Based on “ceRNA hypothesis”, a two-step approach was employed to build context-specific LceNETs. First, the differentially expressed lncRNAs, miRNAs and mRNAs in each group were separately mapped to the global lncRNA-associated ceRNA network to extract context-specific ceRNA networks. Second, expression correlation between lncRNA and mRNA in a candidate ceRNA triplet was evaluated by Pearson correlation coefficient using matched expression profiles. lncRNA-mRNA pairs with R>0.5 and *P*<0.05 were selected as significantly positively correlated interactions, which were then used to construct LceNETs visualized by Cytoscape (v3.6.1) (27). Hub nodes were defined as the top 5% of highest degree nodes in the context-specific LceNET.

### KEGG Pathway Enrichment Analysis of Differentially Expressed mRNAs

To investigate the function of the identified differentially expressed mRNAs, KEGG pathway enrichment analysis was performed using “ClusterProfiler” package (28) in R. Adjusted *P* < 0.05 (Benjamini-Hochberg method) was used as the cut-off for selecting statistically significant KEGG terms. The top five pathways were shown by Circos plots using “GOplot” (29) and “ggplot2” packages in R.

### Bioinformatics Model for lncRNA/miRNA Biomarker Screening

As reported previously, the strength of competitive interaction between lncRNA and mRNA is closely related to the number of common miRNAs shared by two RNA molecules (30). Accordingly, in this study a novel bioinformatics model was applied to screen candidate lncRNA and miRNA biomarkers for colon and rectal cancer. In the model, the NSM measurement was defined to detect the ability of a lncRNA in competitively binding miRNAs in the context-specific ceRNA network. lncRNAs with significantly higher NSM value (*P*<0.05, Wilcoxon signed-rank test) in initiation or metastasis-associated LceNETs were recognized as lncRNA biomarkers. As highlighted in our previous research, the regulatory power of a miRNA can be evaluated by NSR parameter, *i.e.*, the number of single-line regulated-RNAs, since the single-line regulatory site is vulnerable in the network (8). Evidences indicated that miRNAs with high NSR values are likely to be biomarkers (31). Here, we applied NSR in lncRNA-miRNA-mRNA triple network to identify key miRNAs. Those miRNAs with significantly higher NSR value (NSR > average of NSRs and *P*<0.05, Wilcoxon signed-rank test) in initiation or metastasis-associated ceRNA network were recognized as miRNA biomarkers. In the next step, key lncRNA-miRNA-mRNA triplet biomarkers were screened by assessing each component’s function as follows: 1) lncRNA and miRNA were the identified biomarker, respectively; 2) mRNAs were reported as tumor-associated genes (TAGs) or related to the prognosis of colon/rectal cancer. The lncRNA-miRNA-mRNA interactions were visualized by “ggalluvial” package in R.

### ROC Curve Analysis

To evaluate the sensitivity and specificity of the identified lncRNA and miRNA biomarkers for distinguishing between normal and cancer tissues, ROC curve analyses were conducted and the AUC were calculated using the ‘ROCR’ package (32) in R.

### Survival Analysis

To assess the prognostic value of lncRNA/miRNA biomarkers and key mRNAs, patients were divided into high-expression and low-expression groups using the criteria from OncoLnc (http://www.oncolnc.org/) (33). Then, Kaplan-Meier survival analyses were carried out to evaluate the differences in OS times between high-expression and low-expression groups using the “survival” and “survminer” packages in R, and the log-rank test was employed to calculate the statistical significance of the KM survival curves (*P* < 0.05).

### Cell Lines and Culture Condition

CRC cell lines HCT116, SW480, HT29, Lovo and SW620 were obtained from the laboratory of professor Yufeng Xie at the First Affiliated Hospital of Soochow University, and human normal colon mucosal epithelial cell line NCM 460 was purchased from the cell bank of Chinese Academy of Sciences. Cells were cultured in RPMI-1640 medium (HyClone) supplemented with 10% fetal bovine serum (Gibco) at 37°C with 5% CO_2_.

### Quantitative Real-Time Polymerase Chain Reaction (qRT-PCR)

Total RNA was isolated from cultured cells using miRNeasy Mini Kit (Qiagen, Germany) and converted into complementary DNA using 5× All-In-One RT MasterMix (abm, Canada). qRT-PCR was performed in triplicate for each sample using BrightGreen 2× qPCR MasterMix (abm, Canada) and a LightCycler 96 Real-Time PCR detection system (Roche, USA) according to the manufacturer’s instructions. For qRT-PCR, KCNQ1OT1 primers (Sangon Biotech, China) were: 5’-TGCAGAAGACAGGACACTGG-3’ (sense) and 5’-CTTTGGTGGGAAAGGACAGA-3’ (antisense); SNHG1 primers (Sangon Biotech, China) were: 5’-GCCAGCACCTTCTCTCTAAAGC-3’ (sense) and 5’- GTCCTCCAAGACAGATTCCATTTT-3’ (antisense); GAPDH primers (Sangon Biotech, China) were: 5’-GCATCCTGGGCTACACTG-3’ (sense) and 5’-TGGTCGTTGAGGGCAAT-3’ (antisense). The 2-^△△Ct^ method was used to calculate the relative gene expression levels of KCNQ1OT1 and SNHG1, which were normalized to the corresponding *GAPDH* mRNA levels.

## Results

### Construction of the Global lncRNA-Associated ceRNA Network (LceNET) in Human

To facilitate exploring lncRNA-associated competing interactions in cancers, we employed a computational method to uncover genome-wide ceRNA cross-talk in human. Notably, factors including the experiment-supported miRNA-target pairs, the consistency of lncRNA names between ceRNA network and TCGA database, and the miRNA regulatory similarity were considered (see the *Materials* *and* *Methods* section). Based on the identified ceRNA interaction triplets, the global LceNET composed by 3,102 lncRNAs, 1,085 miRNAs and 16,490 mRNAs was constructed in human.

### Characterization of Differentially Expressed RNAs in Colon and Rectal Cancers

**Figure 2:**
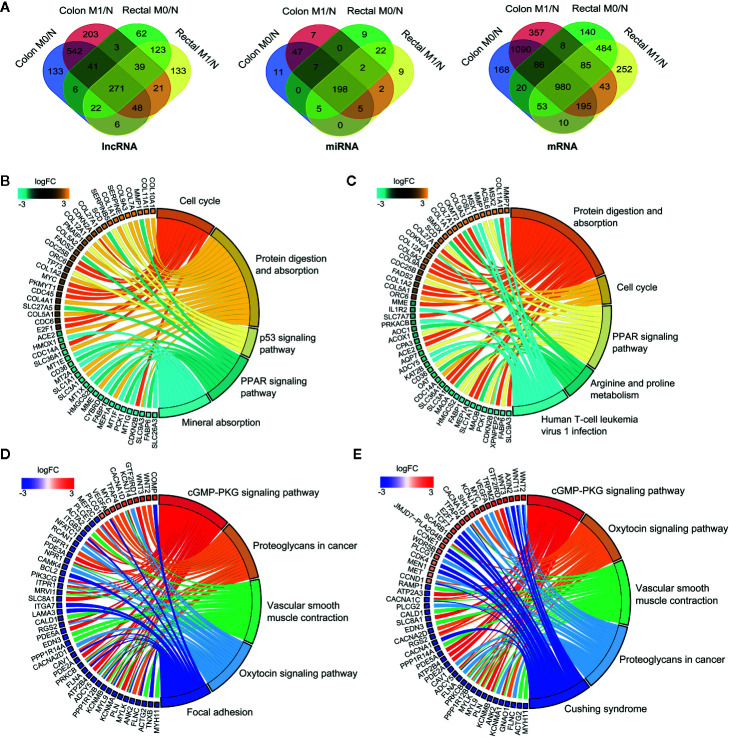
Global landscape of differentially expressed RNAs in colon and rectal cancers. **(A)** Venn diagrams showing the overlapping differentially expressed lncRNAs (left), miRNAs (middle) and mRNAs (right) among colon M0/N, colon M1/N, rectal M0/N and rectal M1/N groups. **(B-E)** The top five significantly enriched KEGG pathways and related genes in colon M0/N **(B)**, colon M1/N **(C)**, rectal M0/N **(D)** and rectal M1/N **(E)** groups, respectively. M0 represents patients without distant metastasis; M1 represents patients with distant metastasis; N represents normal paracancerous tissue.

To systematically elucidate the molecular distinction in the carcinogenesis of colon and rectal cancers, patient samples were divided into without distant metastasis (M0) and with distant metastasis (M1) subgroups from TCGA-COAD and TCGA-READ databases, respectively. We then explored the differential expression profiles of lncRNAs, miRNAs and mRNAs in the four comparable groups, including M0 vs. normal in colon cancer (colon M0/N), M1 vs. normal in colon cancer (colon M1/N), M0 vs. normal in rectal cancer (rectal M0/N) and M1 vs. normal in rectal cancer (rectal M1/N). The number of differentially expressed RNAs (DERNAs) in each comparable group was shown in **Supplementary Table 1-3**. Notably, 271 lncRNAs, 198 miRNAs and 980 mRNAs were identified as common DERNAs in all of the four comparable groups (**Figure 2A**).

To gain insights into the different biological features implicated with colon and rectal carcinogenesis, functional enrichment analysis was respectively performed for differentially expressed mRNAs (DEmRNAs) identified in the above four comparable groups. As shown in **Figures 2B, C**, DEmRNAs identified in colon M0/N and M1/N groups were significantly enriched in diverse critical biological pathways, e.g. “cell cycle”, “protein digestion and absorption”, “p53 signaling pathway” and “PPAR signaling pathway”, revealing their essential roles in tumorigenesis of colon cancer. Comparatively, DEmRNAs in rectal M0/N and M1/N groups were primarily implicated in the well-characterized colorectal cancer-associated pathways including “cGMP-PKG signaling pathway”, “proteoglycans in cancer”, and “vascular smooth muscle contraction” (**Figures 2D, E** **)**. For example, PKG is a promising therapeutic target for metastatic colorectal cancer (34); activating cGMP-PKG signaling pathway could suppress tumor growth by inhibiting Wnt/β-Catenin signaling in colon cancer (35). Notably, DEmRNAs in rectal M0/N and M1/N groups were also significantly enriched in “oxytocin signaling pathway” (**Figures 2D, E** **)**, which exhibits emerging potential links with cancer, but has not been demonstrated to be associated with colorectal cancer previously.

### Dynamical Competitive Interactions Between lncRNAs and mRNAs During the Initiation and Metastasis of Colon and Rectal Cancers

As ceRNA network tightly links the roles of miRNAs with that of coding and non-coding RNAs sharing common MREs, investigating miRNA-mediated ceRNA crosstalk can contribute to elucidate their biological functions in carcinogenesis. Therefore, we set out to establish context-specific LceNETs by extracting lncRNA-miRNA-mRNA interactions through mapping DERNAs from colon M0/N, colon M1/N, rectal M0/N and rectal M1/N groups into the global human LceNETs, respectively.

**Figure 3:**
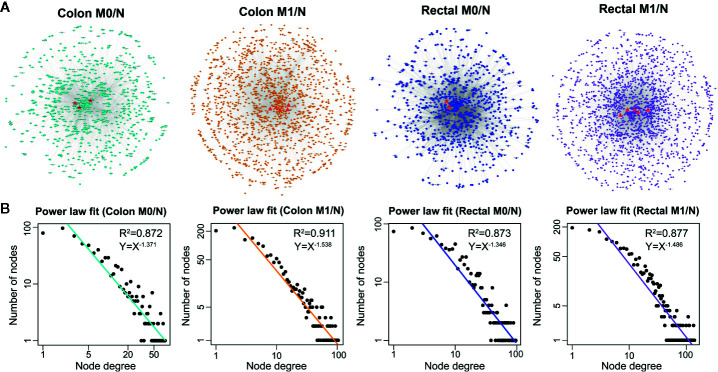
The global view **(A)** and node degree distribution **(B)** of the four context-specific LceNETs. The identified lncRNA biomarkers were highlighted by red triangle. M0 represents patients without distant metastasis; M1 represents patients with distant metastasis; N represents paracancerous normal tissue.

Based on the ceRNA theory, ceRNA triplets consisting of positively correlated lncRNA-mRNA competing pairs were selected. As shown in **Figure 3A**, most of the ceRNA nodes in each LceNET were interconnected and they could cross-talk with each other through shared miRNAs, indicating complex and robust regulatory relationship. Thus, perturbation in key ceRNA interactions may affect the stability of the entire LceNETs. Notably, the sizes of LceNETs associated with metastasis (LceNETs_colon_M1/N and LceNETs_rectal_M1/N) were relatively larger than that implicated with occurrence (LceNETs_colon_M0/N and LceNETs_rectal_M0/N), indicating the complexity of tumor metastasis. In particular, 350 ceRNA interactions were shared by the four networks, suggesting that certain lncRNAs and mRNAs tend to serve as common ceRNAs that regulate the occurrence and metastasis of colon cancer as well as that of rectal cancer (**Supplementary Figure 2**). Besides, it has been shown that various lncRNAs such as MIR22HG and LINC01600 play an important role in colorectal cancer (36, 37). Here, we found that MIR22HG could serve as a ceRNA by sponging miR-25-3p or miR-425-5p in the occurrence and metastasis processes of rectal cancer rather than colon cancer, suggesting its distinct roles in the two cancer types.

Next, topological analysis showed that the degree distribution of nodes in the four LceNETs closely followed a power law distribution with R^2^ > 0.87 (**Figure 3B**), indicating LceNETs were scale-free networks rather than randomly connected networks. As hub nodes with top degree in biological network tend to possess critical function, we investigated the lncRNAs acting as hub nodes in the four LceNETs, respectively. As shown in **Supplementary Table 4**, KCNQ1OT1 and SNHG1 possessed hub nodes properties in all of the four context-specific ceRNA networks, implying significant potential in regulating colorectal carcinogenesis.

### Diagnostic and Prognostic Significance of LceNET-Driven lncRNA Biomarkers Implicated in the Carcinogenesis of Colon and Rectal Cancers

**Figure 4:**
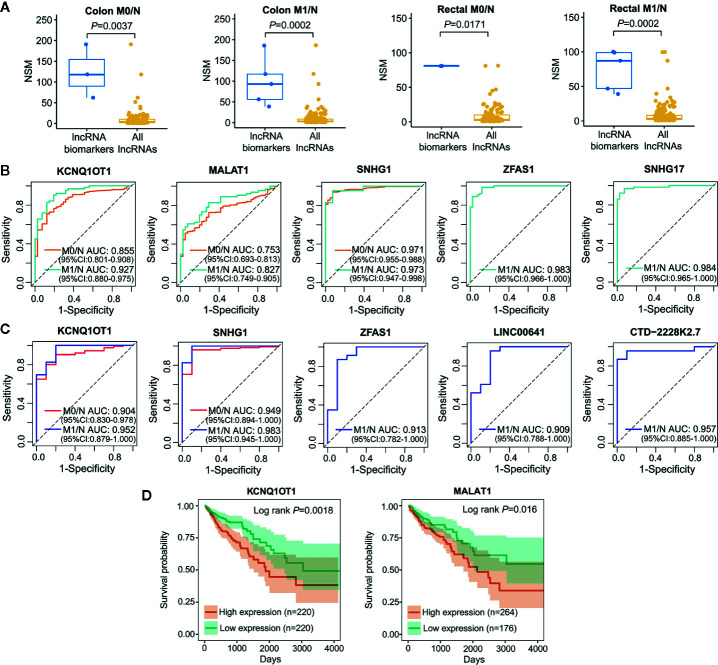
Characterization of lncRNA biomarkers in the initiation and metastasis of colon and rectal cancers. **(A)** NSM distribution of the identified lncRNA biomarkers and all lncRNAs in context-specific ceRNA network. Statistical significance was calculated using the Wilcoxon rank-sum test. **(B, C)** ROC curve analysis was carried out to evaluate the predictive role of lncRNA biomarkers for colon cancer **(B)** and rectal cancer **(C)** in TCGA datasets. **(D)** Kaplan-Meier survival analysis of lncRNA biomarkers that significantly associated with overall survival in colon cancer (n=440) by OncoLnc.

Cancer is a complicated disease with perturbation of various molecular interactions; hence, identifying biomarkers based on systems-guided ceRNA network is urgently needed to provide more reliable and effective signatures for cancer diagnosis and treatment. Here, a computational approach, in which the potential of lncRNA acting as a ceRNA was evaluated by the number of shared miRNAs (NSM), was developed to explore lncRNA biomarkers involved in the initiation and metastasis of colon and rectal cancers based on context-specific LceNETs. As shown in **Figure 4A**, lncRNA biomarkers possessed significantly higher NSM values compared with all lncRNAs in the corresponding context-specific LceNETs. Furthermore, the sensitivity and specificity of lncRNA biomarkers in diagnosing cancer patients without or with distant metastasis were evaluated by receiver operating characteristic (ROC) curve analyses. As shown in **Figures 4B, C**, area under curve (AUC) values of lncRNA biomarkers ranged from 0.75 to 0.97 in distinguishing normal and tumor without distant metastasis, and were more than 0.82 in distinguishing normal and tumor with distant metastasis, revealing superior diagnostic performance.

Notably, lncRNA KCNQ1OT1 and SNHG1 were identified as common biomarkers in colon M0/N, colon M1/N, rectal M0/N and rectal M1/N groups, indicating their critical roles in the carcinogenesis of both colon and rectal cancers; lncRNA MALAT1 was recognized as biomarkers in colon M0/N and colon M1/N groups, suggesting its specific functions in diagnosing colon cancer rather than rectal cancer. Furthermore, lncRNA ZFAS1 was characterized as specific biomarkers in colon M1/N and rectal M1/N groups, indicating it can be used to identify CRC patients with distant metastasis (**Table 1**). To further investigate lncRNA biomarkers playing important roles in cancer prognosis, Kaplan-Meier survival analysis of colon or rectal patients with relatively high or low expression levels of those biomarker were performed, respectively. Strikingly, MALAT1 and KCNQ1OT1 were significantly associated with overall survival (OS) times of colon cancer patients (**Figure 4D**), while lncRNA biomarkers identified in rectal M0/N or M1/N groups were not related to patients prognosis, suggesting they may participate in the regulation of prognosis through other mechanisms.

**Table 1:** lncRNA biomarkers involved in the carcinogenesis of colon and/or rectal cancers.

Groups	lncRNA biomarker
Colon M0/N	MALAT1, KCNQ1OT1 and SNHG1
Colon M1/N	MALAT1, KCNQ1OT1, SNHG1, ZFAS1 and SNHG17
Rectal M0/N	KCNQ1OT1 and SNHG1
Rectal M1/N	KCNQ1OT1, CTD-2228K2.7, SNHG1, ZFAS1 and LINC00641

**Figure 5:**
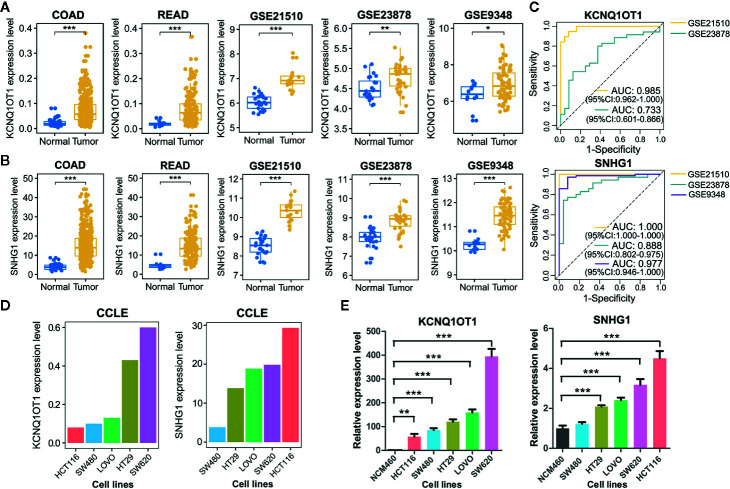
Validation of common lncRNA biomarkers by assessing their expression pattern and diagnostic performance. **(A)** Boxplots showing the expression level of lncRNA KCNQ1OT1 between tumor and normal tissues from TCGA and GEO datasets. **(B)** Boxplots showing the expression level of lncRNA SNHG1 between tumor and normal tissues from TCGA and GEO datasets. **(C)** ROC curve analysis was employed to evaluate the predictive role of KCNQ1OT1 and SNHG1 in GEO datasets. **(D)** Relative expression level of KCNQ1OT1 and SNHG1 in CRC cell lines was detected in the CCLE database. **(E)** Relative expression level of KCNQ1OT1 and SNHG1 in CRC cell lines was determined by qRT-PCR. *P < 0.05, **P < 0.01, ***P < 0.001.

As lncRNAs KCNQ1OT1 and SNHG1 exhibited high potential in diagnosing patients with colon cancer or rectal cancer, we further validated their experimental gene expression pattern and diagnostic performance *via* published GEO datasets including GSE21510, GSE23878, and GSE9348. Notably, both of KCNQ1OT1 and SNHG1 were significantly up-regulated in tumor tissues (**Figures 5A, B** **)** and possessed excellent ability in distinguishing colorectal cancer patients (**Figure 5C**). Consistently, the expression levels of KCNQ1OT1 and SNHG1 were significantly higher in CRC cell lines than that in the normal colonic epithelial cell line NCM460 (**Figure 5E**). Moreover, both of the results from CCLE (Cancer Cell Line Encyclopedia) database and qRT-PCR analysis showed that KCNQ1OT1 and SNHG1 had significantly higher expression level in CRC cell lines with high metastatic potential (e.g. SW620) than that with low metastatic potential (e.g. SW480) (**Figures 5D, E** **)**, implying their roles in promoting CRC metastasis.

### Characterization of miRNA Biomarkers Identified in LceNETs Indicating the Carcinogenesis of Colon and Rectal Cancers

**Figure 6:**
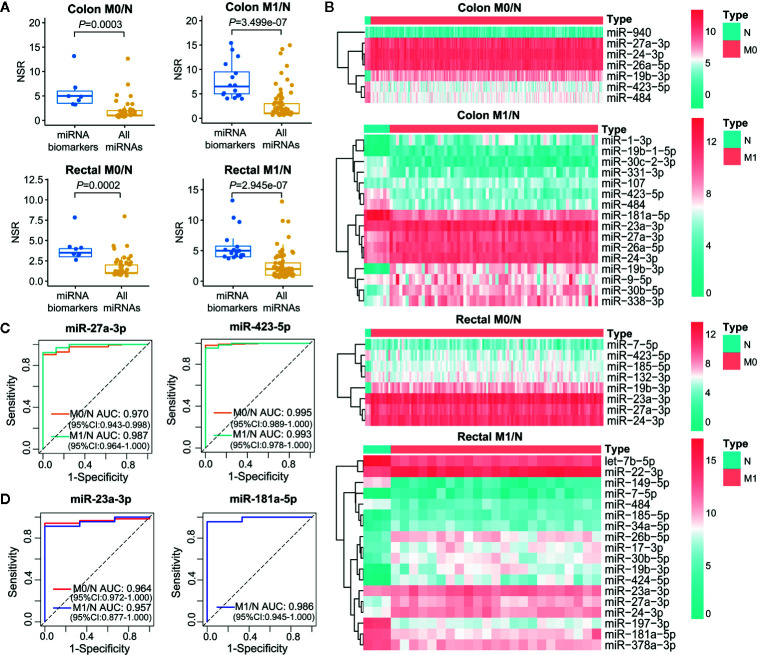
Characterization of miRNA biomarkers in the initiation and metastasis of colon and rectal cancers. **(A)** NSR distribution of the identified miRNA biomarkers and all miRNAs in context-specific ceRNA network. Statistical significance was calculated using the Wilcoxon rank-sum test. **(B)** Heatmap of the expression level of miRNA biomarkers between colon/rectal cancer and normal tissues. M0 represents patients without distant metastasis; M1 represents patients with distant metastasis; N represents paracancerous normal tissue. **(C, D)** ROC curve revealed the clinical role of representative miRNA biomarkers in the screening of colon cancer **(C)** and rectal cancer **(D)**.

Increasing evidence has demonstrated that multiple miRNAs can be used as biomarkers to guide decision on cancer diagnosis and therapy. We employed single-regulatory theory that consider single-line regulated miRNA-target interactions as the vulnerable structure for biological networks, to identify miRNA biomarkers in ceRNA triplet networks for the first time (**Supplementary Table 5**). As shown in **Figure 6A**, miRNA biomarkers possessed significantly higher number of single-line regulation (NSR) values compared with all miRNAs in the corresponding context-specific LceNETs (*P* < 0.05, Wilcoxon rank sum test). Strikingly, miR-27a-3p, miR-24-3p and miR-19b-3p were identified in all of colon M0/N, colon M1/N, rectal M0/N and rectal M1/N groups (**Supplementary Figure 3**), indicating their widely predictive roles in diagnosing colon and rectal cancers.

The different expression level of miRNA biomarkers between tumor and normal tissues could be clearly observed from the heatmaps (**Figure 6B**). Moreover, their excellent distinguishing ability between normal and tumor without or with distant metastasis was validated by ROC curve analysis with all of their AUC values were over 0.95 (**Figures 6C, D** **)**. Unexpectedly, the miRNA biomarkers in neither colon cancer nor rectal cancers were not associated with clinical outcome, suggesting their indirect roles in affecting patient prognosis.

### Key lncRNA-miRNA-mRNA Interactions With Oncogenic Roles Across Colon and Rectal Cancers

**Figure 7:**
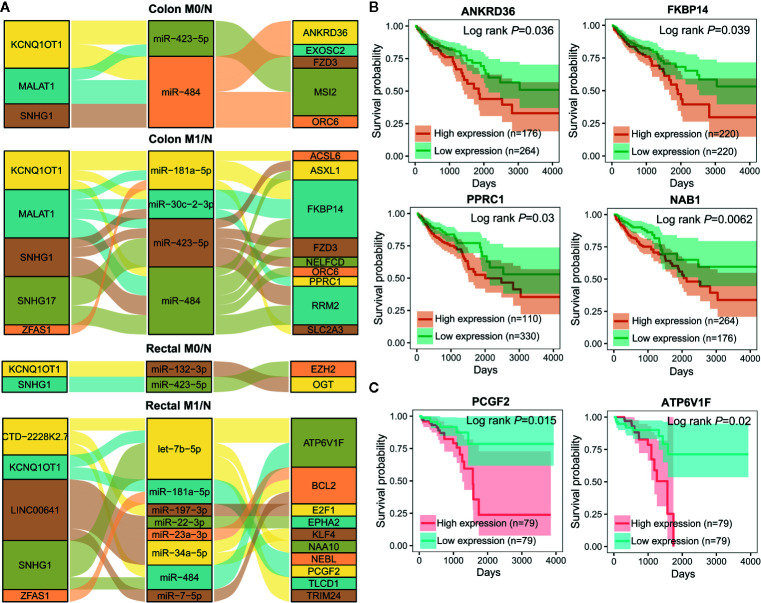
Important lncRNA-miRNA-mRNA interactions composed by lncRNA and miRNA biomarkers and prognostic value of target mRNAs. **(A)** Sankey diagram showing the representative lncRNA-miRNA-mRNA regulatory axes involving lncRNA and miRNA biomarkers. **(B, C)** Kaplan-Meier analysis for overall survival of colon cancer (n=440) **(B)** and rectal cancer (n=158) **(C)** patients according to representative mRNAs expression level by OncoLnc.

To further elucidate the molecular distinction between colon cancer and rectal cancer, key lncRNA-associated competing triplets involved in cancer initiation and metastasis were identified *via* screening interactomes composed by lncRNA and miRNA biomarkers. As miRNA negatively regulate target gene expression, only the triplets in which miRNA and its lncRNA/mRNA target with opposite expression pattern were retained (**Figure 7A**). Importantly, in the identified ceRNA triplets, ANKRD36, FKBP14, PPRC1, and NAB1 were closely related to the prognosis of colon cancer patients (**Figure 7B**), while PCGF2 and ATP6V1F was tightly linked to the prognosis of rectal cancer patients (**Figure 7C**), suggesting that lncRNA biomarker may contribute to CRC occurrence and metastasis by regulating prognosis-related gene expression *via* ceRNA mechanism.

**Figure 8:**
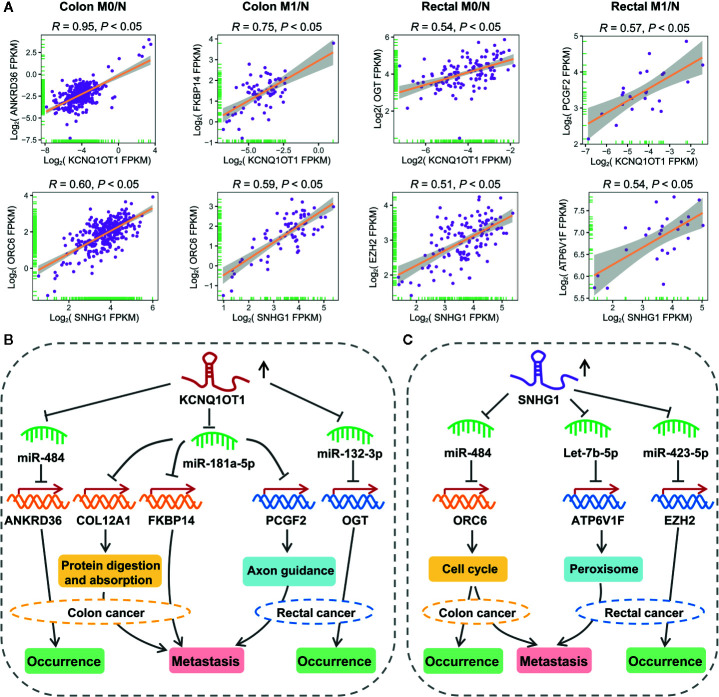
Putative regulatory mechanisms of KCNQ1OT1 and SNHG1 underlying the initiation and metastasis of colon and rectal cancers. **(A)** Expression correlation of KCNQ1OT1 and its ceRNAs analyzed by Pearson correlation coefficient. We use the non-log scale for calculation and use the log-scale axis for visualization. **(B)** Expression correlation of SNHG1 and its ceRNAs analyzed by Pearson correlation coefficient. We use the non-log scale for calculation and use the log-scale axis for visualization. **(C)** Distinct mechanism of KCNQ1OT1 and SNHG1 in the processes of initiation and metastasis of colon and rectal cancers.

As shown in **Figure 7A**, lncRNA KCNQ1OT1 and SNHG1 may contribute to the progression of colon and rectal cancers through distinct mechanisms. For example, KCNQ1OT1/miR-484/ANKRD36, KCNQ1OT1/miR-181a-5p/FKBP14 or COL12A1, KCNQ1OT1/miR-132-3p/OGT, and KCNQ1OT1/miR-181a-5p/PCGF2 regulatory axes were respectively identified in colon M0/N, colon M1/N, rectal M0/N and rectal M1/N groups. Similarly, SNHG1/miR-484/ORC6 was discovered in both of colon M0/N and colon M1/N groups, while SNHG1/miR-423-5p/EZH2 and SNHG1/let-7b-5p/ATP6V1F were identified in rectal M0/N and rectal M1/N groups, respectively. Furthermore, the correlation between lncRNAs and representative target mRNAs in the identified ceRNA triplets were examined. As expected, KCNQ1OT1 strongly correlated with ANKRD36, FKBP14, OGT, and PCGF2, and SNHG1 strongly correlated with ORC6, ORC6, EZH2, and ATP6V1F in colon M0/N, colon M1/N, rectal M0/N, and rectal M1/N groups, respectively (**Figure 8A**). By analyzing the significantly enriched KEGG pathways, we found that COL12A1 and PCGF2 regulated by KCNQ1OT1, were closely involved in “Protein digestion and absorption” and “axon guidance” pathways, respectively. Comparatively, ORC6 and ATP6V1F were enriched in “cell cycle” and “Peroxisome” pathways, respectively. Based on the above results, we proposed the possible regulatory mechanisms of KCNQ1OT1 and SNHG1 acting as ceRNA in colon cancer and rectal cancer (**Figures 8B, C** **)**.

## Discussion

As reported, the dysregulated lncRNAs could contribute to tumor initiation and progression through a ceRNA mechanism by competitively sponging shared miRNAs with their target genes (38, 39). Recently, researchers tend to investigate lncRNA biomarkers in various cancers based on ceRNA network, which mainly include two steps: 1) identifying differentially expressed lncRNAs/miRNAs/mRNAs; 2) discovering miRNA-mRNA and miRNA-lncRNA pairs composed by differentially expressed lncRNAs/miRNAs/mRNAs *via* computational algorithms or experimental methods. For example, Gao et al. (40) unveiled lncRNA, miRNA and mRNA prognostic biomarkers associated with invasive breast cancer; Zhang G. et al. (41) identified new panel biomarkers indicating the occurrence and recurrence of myocardial infarction. We herein initially constructed a global LceNET in human and collected miRNA-mRNA and miRNA-lncRNA interactions verified by experimental methods, e.g. luciferase reporter assay, HITS-CLIP and PAR-CLIP, from the most commonly used databases. Then a novel computational model was developed and applied to uncover lncRNA biomarkers based on context-specific LceNET by considering their miRNA binding ability.

Furthermore, efforts have been made to comparatively investigate the heterogeneity in cancer-related pathways between colon and rectal cancer. Strikingly, DEmRNAs identified in colon cancer were significantly enriched in pathways, e.g. “cell cycle” (42), “protein digestion and absorption” (43), “p53 signaling pathway” (44) and “PPAR signaling pathway” (45), while DEmRNAs in rectal cancers were closely implicated in the pathways including “cGMP-PKG signaling pathway” (46), “proteoglycans in cancer” (47), and “vascular smooth muscle contraction” (48) and “oxytocin signaling pathway” (**Figures 2B–E**), indicating distinct oncogenic mechanisms of colon and rectal cancers. Especially, among those top enriched pathways, “oxytocin signaling pathway” is newly deciphered to be associated with colorectal cancer.

Based on our computational model, KCNQ1OT1 and SNHG1 possessing hub nodes properties in all of the four context-specific ceRNA networks, were identified as shared lncRNA biomarkers in colon and rectal cancer, revealing their critical role in colorectal carcinogenesis. Consistently, both of KCNQ1OT1 and SNHG1 have been demonstrated as oncogenes through ceRNA mechanisms in CRC. For example, KCNQ1OT1 has been reported to promote drug resistance of CRC cells by sponging miR-34a (49) or miR-760 (50), and contribute to cell proliferation, migration and EMT formation in CRC through regulating miR-217/ZEB1 axis (51); SNHG1 could act as decoy of miR-137 (52), miR-497 (53), miR-195-5p (53), miR-154-5p (54) and miR-145 (55) to weaken their suppressive effect on target genes, thereby facilitating colorectal tumorigenesis.

As an interesting result, we found that KCNQ1OT1 and SNHG1 could regulate the initiation and metastasis of colon and rectal cancers through distinct ceRNA regulatory axes, which have not been reported in previous studies. As shown in the proposed mechanism, KCNQ1OT1 may regulate the initiation of colon cancer by sponging miR-484 to derepress its inhibiting effect on ANKRD36 that related to prognosis, accelerate the initiation of rectal cancer by acting as a miR-132-3p-mediated ceRNA of OGT, while promote the metastasis of colon and rectal cancers through miR-181a-5p-mediated ceRNA regulatory relationship (**Figure 8B**). In addition, SNHG1 exhibited potentials in regulating the initiation and metastasis of colon cancer *via* cell cycle signaling pathway by working as a miR-484-mediated ceRNA of ORC6. Comparatively, SNHG1 was found to promote initiation and metastasis of rectal cancer through regulating let-7b-5p/ATP6V1F and miR-423-5p/EZH2 axes, respectively (**Figure 8C**). Especially, serum miR-423-5p and miR-484 have been proven as diagnostic biomarkers for CRC (56). Taken together, these results have shown distinct mechanism between colon and rectal cancers in terms of initiation and metastasis.

Although SNHG1 or KCNQ1OT1 has been reported to be significantly correlated with the prognosis of colon cancer (57–61), the regulatory mechanism of SNHG1 or KCNQ1OT1 underlying the pathogenic differences between colon cancer and rectal cancer has not been well explored. Hence, our findings provide insights into the oncogenetic heterogeneity between colon and rectal cancers. In addition, MALAT1, ZFAS1 and SNHG17 have been identified as diagnostic biomarker specific to colon cancer, the metastasis of colon and rectal cancer, and the metastasis of colon cancer, respectively, therefore the tumor/stage-specific roles of MALAT1, ZFAS1 and SNHG17 in colon and rectal cancers need further investigation.

## Conclusion

In summary, we constructed context-specific LceNETs that reveal distinct mechanisms underlying the initiation and metastasis between colon and rectal cancer. A novel computational model was proposed and applied for lncRNA biomarker identification, which will greatly facilitate systematic investigation of LceNETs in various human cancers. Moreover, we found that different pathways, lncRNA biomarkers and miRNA biomarkers were closely involved in the tumorigenesis between colon and rectal cancer. Especially, the KCNQ1OT1 and SNHG1 were unveiled as common lncRNA biomarkers with critical roles in the initiation and metastasis of colon and rectal cancers *via* distinct ceRNA mechanisms.

## Data Availability Statement

Publicly available datasets were analyzed in this study. This data can be found here: http://cancergenome.nih.gov/, https://www.ncbi.nlm.nih.gov/geo/.

## Author Contributions

BS and XQ designed the research. YL developed the computational method. XQ and XL collected the data and performed the computational and experimental analyses. XQ, YL, XL, JC, and BS drafted and revised the manuscript. BS supervised and coordinated the research. All authors contributed to the article and approved the submitted version.

## Funding

This work was supported by National Natural Science Foundation of China (Grant Nos. 31900490, 31770903, and 31670851), Jiangsu Planned Projects for Postdoctoral Research Funds (2018K173C), China Postdoctoral Science Foundation (2019M651938) and the Natural Science Foundation of the Jiangsu Higher Education Institutions of China (19KJB180027).

## Conflict of Interest

The authors declare that the research was conducted in the absence of any commercial or financial relationships that could be construed as a potential conflict of interest.
