# Supplementary material for: Biomarker Discovery for the Carcinogenic Heterogeneity Between Colon and Rectal Cancers Based on lncRNA-Associated ceRNA Network Analysis
Source: Front Oncol. 2020 Oct 30;10:535985. doi: 10.3389/fonc.2020.535985 (PMC7662689; doi:10.3389/fonc.2020.535985)
Supplement: Supplementary file 1 [file DataSheet_1.pdf]

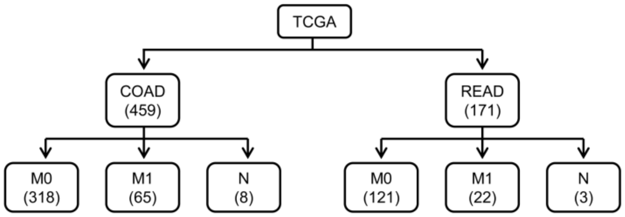

Supplementary Figure 1. Sample number of each group investigated in the present study.

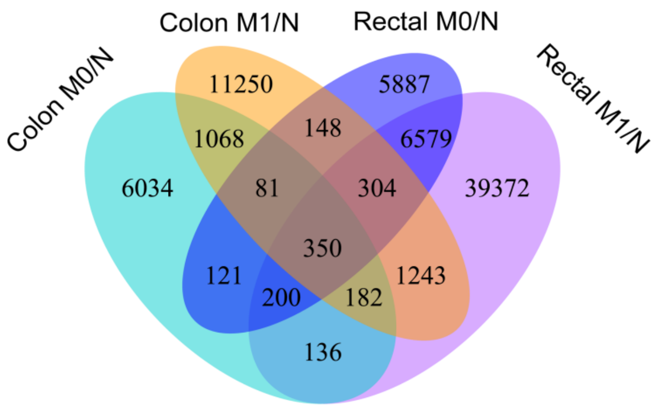

Supplementary Figure 2. Venn diagram for the overlapping ceRNA interactions in the four ceRNA networks.

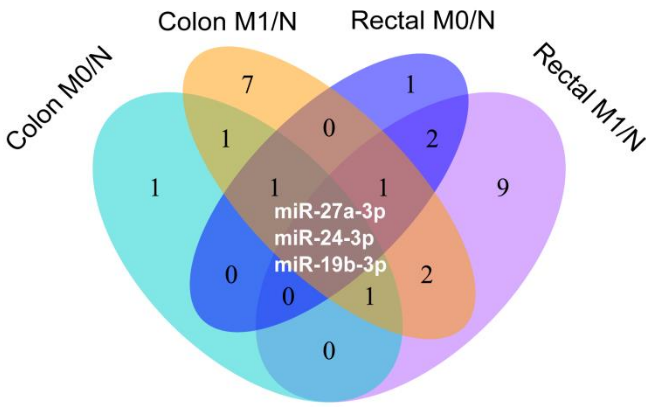

Supplementary Figure 3. Venn diagram for the overlapping miRNA biomarkers in the four ceRNA networks.
